# Elevated PINK1/Parkin-Dependent Mitophagy and Boosted Mitochondrial Function Mediate Protection of HepG2 Cells from Excess Palmitic Acid by Hesperetin

**DOI:** 10.1021/acs.jafc.3c09132

**Published:** 2024-05-29

**Authors:** Wan Li, Zhengnan Cai, Florian Schindler, Leila Afjehi-Sadat, Bianca Montsch, Petra Heffeter, Elke H. Heiss, Wolfram Weckwerth

**Affiliations:** † Molecular Systems Biology (MOSYS), Department of Functional and Evolutionary Ecology, University of Vienna, Vienna 1030, Austria; ‡ Vienna Doctoral School of Ecology and Evolution, University of Vienna, Vienna 1030, Austria; § Vienna Doctoral School of Pharmaceutical, Nutritional and Sports Sciences, University of Vienna, Vienna 1090, Austria; ∥ Mass Spectrometry (Core) Facility, University of Vienna, Vienna 1030, Austria; ⊥ Research Support Facilities UBB, University of Vienna, Vienna 1030, Austria; # Center for Cancer Research and Comprehensive Cancer Center, Medical University of Vienna, Vienna 1090, Austria; ∇ Department of Food Chemistry and Toxicology, University of Vienna, Vienna 1090, Austria; ○ Department of Pharmaceutical Sciences, University of Vienna, Vienna 1090, Austria; ◆ Vienna Metabolomics Center (VIME), University of Vienna, Vienna 1030, Austria

**Keywords:** hesperetin, mitochondrial dysfunction, metabolomics, PINK1/Parkin-mediated mitophagy degradation, TCA cycle and fatty acid oxidation

## Abstract

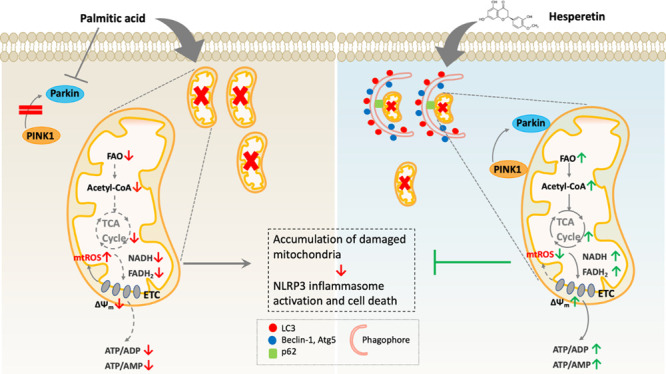

Deregulation of mitochondrial functions in hepatocytes contributes to many liver diseases, such as nonalcoholic fatty liver disease (NAFLD). Lately, it was referred to as MAFLD (metabolism-associated fatty liver disease). Hesperetin (Hst), a bioactive flavonoid constituent of citrus fruit, has been proven to attenuate NAFLD. However, a potential connection between its preventive activities and the modulation of mitochondrial functions remains unclear. Here, our results showed that Hst alleviates palmitic acid (PA)-triggered NLRP3 inflammasome activation and cell death by inhibition of mitochondrial impairment in HepG2 cells. Hst reinstates fatty acid oxidation (FAO) rates measured by seahorse extracellular flux analyzer and intracellular acetyl-CoA levels as well as intracellular tricarboxylic acid cycle metabolites levels including NADH and FADH_2_ reduced by PA exposure. In addition, Hst protects HepG2 cells against PA-induced abnormal energetic profile, ATP generation reduction, overproduction of mitochondrial reactive oxygen species, and collapsed mitochondrial membrane potential. Furthermore, Hst improves the protein expression involved in PINK1/Parkin-mediated mitophagy. Our results demonstrate that it restores PA-impaired mitochondrial function and sustains cellular homeostasis due to the elevation of PINK1/Parkin-mediated mitophagy and the subsequent disposal of dysfunctional mitochondria. These results provide therapeutic potential for Hst utilization as an effective intervention against fatty liver disease.

## 1 Introduction

Excess free fatty acids (FFAs) have been well-established to be associated with metabolic syndrome, nonalcoholic fatty liver disease (NAFLD), obesity, and diabetes.^1−3^ In hepatocytes, FFAs undergo re-esterification and incorporate into lipid droplets for storage, or are metabolized through mitochondrial β-oxidation (FAO).^4^ When the supply of FFAs exceeds the hepatic capabilities of handling them, excessive FFAs accumulate in hepatocytes, impair FAO, and lead to oxidative stress, which can deteriorate mitochondrial functions.^5^ Recent studies have highlighted that hepatocellular injury is characterized by dysfunctional mitochondrial activities.^6,7^ Malfunctional mitochondria are deficient in producing ATP and other biosynthetic precursors. In addition, the accumulation of defective mitochondria aggravates reactive oxygen species (ROS) production, which in turn induces the downstream activation of NLRP3 inflammasome.^8,9^ The NLRP3 inflammasome, a multiprotein complex, serves as a platform to promote the cleavage of pro-caspase-1 into its active form caspase-1, and subsequently the maturation of IL-1β and IL-18. IL-1β and IL-18 stimulate systemic inflammation responses and ultimately lead to cell damage and liver injury.^10,11^ Palmitic acid (PA) is the most abundant saturated fatty acid in human plasma, constituting 20–30% of the total fatty acids. Elevated intracellular levels of PA can lead to the production of harmful lipid intermediates such as diacylglycerol and ceramide, which can impair the endoplasmic reticulum and mitochondria, activate inflammatory pathways, and promote apoptosis and oxidative stress.^12^

As the central powerhouses of the cells, mitochondria produce ATP to meet cellular energy requirements through the electron transport chain in their inner membranes. This process can be accompanied by superoxide formation.^13^ Constant exposure to high concentrations of ROS makes mitochondria particularly susceptible to oxidative damage, causing their structural and functional failure.^14,15^ Therefore, cells employ a specific form of macroautophagy to selectively target and degrade abnormal mitochondria, which is known as mitophagy.^16^ Efficient mitophagy contributes to mitochondrial quality control and plays a fundamental role in tissues with a high need for ATP and dependence on mitochondrial energy production, such as the brain, heart, liver, and kidney.^17−19^ Several previous studies have shown that mitophagy induction in hepatocytes enables the maintenance of adequate functional mitochondria and meets cellular energy demands, prevents FFAs-induced hepatocyte injury, and eventually alleviates the development of NAFLD.^6,20^ As such, targeting mitochondrial function appears to be a viable approach to prevent liver injury and relieve the progression of NAFLD.

Hesperetin (Hst), a flavanone that bears a methoxy group at the 4′ position, is abundantly found in sweet oranges, lemons, and grapefruits. Generally, as an important bioactive citrus flavonoid, Hst has higher bioavailability than hesperidin (Hsd), which complements antioxidative and anti-inflammatory properties, leading to its strong hepatoprotective potential.^21,22^ It is well-accepted that inflammation and oxidative stress are tightly correlated with the loss of hepatocytes and normal liver functions, involved in most forms of liver pathology.^22^ Based on that, more and more attention has focused on the hepatoprotective role that Hst plays in the prevention of NAFLD.^23,24^ Indeed, recent studies have revealed that Hst has beneficial effects on NAFLD amelioration as a result of reduced FFAs-induced hepatic oxidative damage, inflammation, and cell apoptosis. Additionally, there is substantial evidence that mitophagy defects are another major contributor to hepatocellular injury in the progression of NAFLD.^25,26^ PTEN-induced putative kinase1 (PINK1) and Parkin have been proposed to be key regulators of mitophagy.^27^ However, whether the inhibition of FFAs-induced hepatic cell damage by Hst is associated with PINK1/Parkin-driven mitophagy remained unresolved. In the present work, we set out to understand the mechanism by which Hst protects hepatocytes against PA-stimulated cellular injury in HepG2 cells. Our results have confirmed that the protective properties of Hst were achieved by facilitating mitochondrial function and promoting PINK1/Parkin-mediated mitophagy.

## 2 Materials and Methods

### 2.1 Reagents and Materials

Hesperetin (HY-N0168, purity ≥98%), cyclosporin A (CsA HY-B0579), JC-1 kits (HY-K0601), oligomycin (HY-N6782), rotenone (HY-B1756), FCCP (HY-50202), BPTES (HY-12683), etomoxir (HY-50202), UK5099 (HY-15475), and CCK-8 (HY-K0301) were obtained from MedChemExpress, New Jersey. l-glutamine solution (25030081), TrypLE Express Enzyme (12604021), and DMEM (31053028) were obtained from Gibco in Grand Island. Palmitic acid (P5585), antimycin (A8674), and 4′,6-diamidino-2-phenylindole (DAPI) (MBD0015) were purchased from Sigma-Aldrich in St. Louis, Missouri. All other reagents were of analytical grade and were obtained from Sigma-Aldrich in St. Louis, Missouri.

### 2.2 Preparation of PA Stocking Solution, Cell Culture, and Treatments

Preparation of the PA stocking solution: PA was prepared in 0.1 M NaOH solution and incubated at 70 °C and 700 rpm/min for 20 min on a thermo shaker until fully dissolved. Then, it was slowly added into 10% fatty-free bovine serum albumin (BSA) solution and rotated for 30 min at 40 °C (700 rpm/min on thermo shaker) to prevent PA precipitation. BSA was prepared in phosphate-buffered saline (PBS) and centrifuged at 10,000*g* for 20 min. The 20 mM PA stock solution was adjusted to pH 7.4 and kept at −80 °C.

HepG2 cells were obtained from American Type Culture Collection (ATCC) and cultured in Dulbecco’s modified Eagle’s medium (DMEM) medium supplemented with 10% heat-inactivated fetal bovine serum (FBS), 2 mM l-glutamine, 100 U/mL penicillin, and 100 μg/mL streptomycin. For the metabolomic analysis, dialyzed heat-inactivated FBS (A3382001, Gibco) was used. For treatments, a 10 mM Hst stocking solution was prepared in dimethyl sulfoxide (DMSO) and stored at −80 °C. HepG2 cells were pretreated with either 10% BSA and 0.1% DMSO (set as the control group) or different concentrations of Hst (20 and 40 μM with 0.1% DMSO) for 4 h and then stimulated with 400 μM PA for 24 h. In the experiment with the CsA, HepG2 cells were preincubated with CsA at a concentration of 5 μM for 4 h prior to treatment with Hst and PA.

### 2.3 Cell Viability

1.2 × 10^5^ cells/well of HepG2 cells were seeded onto 6-well plates and incubated under 37 °C, 5% CO_2_ overnight. Then, Hst solution was added into each well at concentrations of 10, 20, 40, 80, and 100 μM, respectively. After 24 h of incubation, the number of viable cells and cell viability were measured using the Countess II Automated Cell Counter (Invitrogen) and CCK-8 kit according to the manufacturer’s instructions. For the number of viable cells measurement, in brief, after treatments, the cells were washed and resuspended in the medium; 10 μL of the cell suspension was thoroughly mixed with 10 μL of trypan blue (T10282, Invitrogen) and added onto cell counting chamber slides (Invitrogen). The results were analyzed with the Countess II Cell Counter. To estimate the cytoprotective effects of Hst on PA-triggered cell death, HepG2 cells were pretreated with or without Hst (20 and 40 μM) for 4 h, then stimulated with PA for 24 h; the percentage of cell death and cell viability were determined by Countess II Cell Counter and CCK-8 kit, respectively. For the CCK-8 assay, HepG2 cells were seeded onto 96-well plates at a density of 5 × 10^3^ cells/well and incubated overnight. After treatment, the media were discarded and replaced with 100 μL of fresh DMEM containing 10% CCK-8 for each well. Incubation for 1 h, the absorbance was read by a microplate reader (Thermo Scientific) at 450 nm.

### 2.4 Mitochondrial Membrane Potential

The mitochondrial membrane potential was determined by JC-1 staining. HepG2 cells were seeded onto confocal dishes (734-2904, VWR) at 8 × 10^5^ cells/well and incubated for 24 h. After different treatments, 1 mL of PBS containing the final concentration of JC-1 staining solution at 2 μM was added into each well and incubated for 20 min under 37 °C. Then the cells were washed three times with PBS. Hoechst 33342 was used to stain the nuclei. Images were taken on a confocal laser scanning microscope (LSM700, Carl Zeiss) equipped with a 63 Å/1.4 oil differential interference contrast M27 objective lens (Plan Apochromat, Carl Zeiss) and processed by Zeiss ZEN2.5 software.

### 2.5 Measurement of Mitochondrial ROS

HepG2 cells at a density of 1 × 10^6^ cells/well were seeded onto confocal glass-bottom dishes and incubated for 24 h followed by different treatments. The mtROS was visualized and analyzed using mitoSOX Red Mitochondrial Superoxide Indicator (M36008, Invitrogen). The cells were incubated with 1 μM mitoSOX Red and Hoechst 33342 for 30 min under 37 °C and washed three times with PBS. Images were collected on a confocal microscope. The intensity was subsequently measured using the Zeiss ZEN2.5 software.

### 2.6 Spinning Disk Microscopy

HepG2 cells at a density of 1 × 10^5^ cells/well were seeded onto confocal glass-bottom dishes and incubated for 24 h followed by PA and Hst treatments for another 24 h. Subsequently, the cells were incubated with ProlongLife at a dilution of 1:100 (P36974, Invitrogen) for 1 h at 37 °C in a CO_2_ incubator. Then, the medium was discarded and incubated with 250 nM MitoTracker Green (M7514, Invitrogen) for 30 min. Afterward, Hoechst 33342 was added in cells at a 1:100 dilution before measurement to serve as a counterstain for nuclei. In the end, z-stacks of the living cells were acquired by using an Olympus IXplore SpinSR spinning disk confocal microscope. In total, three pictures per spot were obtained.

### 2.7 Detection of Oxygen Consumption Rate (OCR)

OCR was measured by using a Seahorse XFe24 analyzer (Seahorse Bioscience, USA) according to previous protocols.^28^ Briefly, HepG2 cells were seeded onto 6-well plates at 5 × 10^5^ cells per well. After treatments as described before, cells were collected and plated into XF24e-cell culture plates precoated with cell-tak (35420, Corning) for proper adhesion with XF assay medium (103575, Agilent Technologies) supplied with glutamine, pyruvate, and glucose. To hydrate the XF extracellular flux sensor cartridge, each well of a utility plate was filled with 1 mL calibrant solution and incubated in a non-CO_2_ incubator at 37 °C overnight. After treatment, the cells were placed in a non-CO_2_ incubator for 1 h. Then, the real-time OCRs were measured by the seahorse analyzer. Oligomycin (3 μM), FCCP (3 μM), and rotenone/antimycin A (0.5 μM) were injected into the ports of A, B, and C. For fatty acid oxidation (FAO) experiments, cells were incubated in the XF assay medium supplied with glutamine, pyruvate, glucose, and palmitate-conjugated BSA. Etomoxir (4 μM) and BPTES/UK5099 (2 μM) were added to each well. Fatty acid dependency of OXPHOS was analyzed by Wave Software (Agilent Technologies).

### 2.8 Immunofluorescence Staining

HepG2 cells were plated in confocal dishes at a density of 2 × 10^5^ cells/well overnight. Upon treatment, the cells were fixed with cold 4% PFA in PBS (VWR, T61899-AK) for 20 min and then incubated with 0.5% Triton X-100 in PBS for 5 min to permeabilize cells. To block nonspecific binding, the cells were treated with 1% BSA at room temperature for 1 h. Subsequently, the cells were incubated with antibodies against LC3 (1:200), PINK1 (1:300), Parkin (1:400), and Tom 20 (1:400) in combination or separately at 4 °C overnight. Washing three times with tris-buffered saline, 0.1% Tween 20 (TBST), the cells were probed with corresponding secondary antibodies (1:500). Nuclear counterstaining was used in DAPI (1:400) incubation for 5 min. The images were acquired and analyzed by a confocal microscope as described before. The antibodies mentioned were anti-LC3 (sc-398822) and anti-PINK1 (sc-517353) were purchased from Santa Cruz (Heidelberg, Germany), anti-Tom 20 (42406) were bought from Cell Signaling (Danvers, USA), and anti-Parkin (66674-1-Ig), CoraLite 488-conjugated goat anti-mouse IgG (H+L) (SA00013-1) and CoraLite 594-conjugated goat anti-rabbit IgG (H+L) (SA00013-4) were obtained from Proteintech Group (Munich, Germany).

### 2.9 RT-qPCR Analysis

HepG2 cells were seeded in 6-well plates at a concentration of 1 × 10^6^ cells/well overnight. After different treatments, cells were washed twice with cold PBS. TRI reagent was added in cells and total RNA was isolated according to the manufacturer’s instructions. The concentration and quality of RNA were determined using Nanodrop (Thermo Fisher Scientific). Then, 1 μg of total RNA was reverse transcribed using the GoScript TM Reverse Transcription Kit (Promega). The Luna Universal qPCR Master Mix (New England Biolabs; M3003E) was performed to evaluate the mRNA levels of target genes on a CFX96 real-time system. Relative mRNA expression levels were determined by the comparative Ct (2-ΔΔCt) method. Actin gene was taken as the internal control. The primer sequences used are as follows: IL-1β (forward primer: 5′-CTGTCCTGCGTGTTGAAAGA-3′, reverse primer: 5′-TTGGGTAATTTTTGGGATCTACA-3′), IL-18 (forward primer: 5′-TGCATCAACTTTGTGGCAATGA-3′, reverse primer: 5′-GTCCGGGGTGCATTATCTCT-3′), actin (forward primer: 5′-CCAACCGCGAGAAGATGA-3′, reverse primer: 5′-TCCATCACGATGCCAGTG-3′).

### 2.10 Enzyme-Linked Immunosorbent Assay (ELISA) for IL-1β

HepG2 cells (5 × 10^5^ cells/well) were seeded in a 12-well plate overnight. Upon different treatments for 24 h, cell-free supernatants were collected and centrifuged. The release of IL-1β was assessed using an ELISA kit (KE00021, Proteintech), following the manufacturer’s instructions. The experiment was performed in three biological replicates.

### 2.11 Immunoblotting

Cells were lyzed in cold RIPA lysis buffer, which contains protease inhibitor cocktails (Sigma, 4693116001), followed by sonication and centrifugation at 21,000*g* for 15 min at 4 °C. All samples were performed on ice and the concentrations of protein samples were measured by the BCA kit (Millipore, 71285-3). The normalized proteins (10 μg per lane) were dissolved in laemml sample buffer and separated by 8–12.5% SDS PAGE. After being transferred onto PVDF and blocked with 5% low-fat milk at RT for 1 h, membranes were then incubated with primary antibodies at 4 °C overnight. Afterward, the membranes were washed and incubated with peroxidase-labeled secondary antibodies. The bands were visualized by the ECL system (Cytiva, GERPN2232) and acquired by an iBrightFL1500 Imaging System (Invitrogen). The signal intensity was analyzed by ImageJ. The antibodies used were anti-caspase-1 (sc-56036), anti-NLRP3 (sc-134306), anti-Atg5 (sc-133158), anti-LC3 (sc-398822), and anti-cytochrome C (sc-13156) were purchased from Santa Cruz (Heidelberg, Germany), anti-PINK1 (6946), p62 (5114), Beclin-1 (3495), Tom 20 (42406), and PDHA1 (3205) were obtained from Cell Signaling (Danvers, USA), anti-Tim 23 (11123-1-AP), anti-Parkin (66674-1-Ig), anti-α-Tubulin (1224-1-AP), horseradish-peroxidase (HRP)-conjugated anti-rabbit Ig(H + L) (SA00001-2), and (HRP)-conjugated anti-mouse Ig(H + L) (SA00001-1) were obtained from Proteintech Group (Munich, Germany).

### 2.12 siRNA Transfection

HepG2 cells were cultured onto 6-well plates at a density of 5 × 10^5^ cells/well. Until 50% confluence, cells were transfected with control-siRNA or *Pink1* siRNA using lipofectamine 3000 Transfection Reagent (Invitrogen). Briefly, 8 μL of Lipofectamine 3000 was diluted with 100 μL of Opti-MEM and incubated at room temperature. This mixture was then combined with 8 μL of siRNA (final concentration 100 nM), which was also diluted in 100 μL of Opti-MEM. After 5 min, the solutions were mixed gently, and the incubated mixture was allowed to stand for 15 min at room temperature. Meanwhile, the cells were washed twice with PBS and 0.8 mL of fresh medium was added to each well. The transfection mixture was then immediately added to the cells and incubated for 8 h at 37 °C in a CO_2_ incubator. After 48 h of transfection, the cells were treated and processed, as described above. *Pink1* siRNA (sc-44598) and control-siRNA (sc-37007) were obtained from Santa Cruz (Heidelberg, Germany).

### 2.13 Metabolomic Analysis by GC–MS and LC–MS

For metabolomic analysis, dialyzed heat-inactivated FBS was used for cell culture. Cellular metabolites were extracted and analyzed according to previous established methods with modifications.^29^ Briefly, HepG2 cells were seeded at 1 × 10^6^ cells per well of a 6-well plate and allowed to attach overnight. Cells were then preincubated with 40 μM Hst for 4 h before being treated with 400 μM PA for 24 h. After treatment, cells were washed in cooled 0.9% NaCl twice and extracted in 1 mL of 80% methanol (−20 °C) with 2.5 nM phenyl β-d-glucopyranoside as the internal extraction standard. Extraction samples were incubated for 30 min at 4 °C and then centrifuged for 10 min at 21,000*g*. The supernatants were transferred to new Eppendorf tubes and dried in a SpeedVac system (Labogene). The cell pellets were lysed by RIPA and used to measure protein levels for normalization purposes. 15 μL of methoxyamine hydrochloride solutions (40 mg dissolved in 1 mL pyridine) were added to the dried fraction and prepared QC pools, which were then incubated for 90 min at 30 °C. Subsequently, 60 μL of *N*-methyl-*N*-trimethylsilyltrifluoroacetamid (MSTFA) was added and incubated for 30 min at 37 °C. Reaction mixtures were centrifuged for 10 min and 4 °C at 21,000*g* and the supernatant was transferred to a glass vial with microinsert. Measurement of metabolites was performed using the established gas chromatography–mass spectrometry (GC–MS) standard procedure.^30^ 1 μL of the sample was injected at a 1:5 split ratio. Deconvolution of the total ion chromatogram, peak alignment, and integration was performed using the software MS-DIAL v4.9.^31^

The analysis of cellular acetyl-CoA, NAD^+^, NADH, FAD ATP, ADP, and AMP was performed using an UltiMate 3000 UHPLC (Thermo Fisher Scientific) system coupled to an LTQ-Orbitrap Elite mass spectrometer (Thermo Fisher Scientific). HepG2 were seeded at 1 × 10^6^ cells per well of a 6-well plate and allowed to attach overnight. Cells were pretreated with 40 μM Hst in the absence or presence of 400 μM PA. After another 24 h incubation, cells were washed in cooled 0.9% NaCl twice and extracted in 1 mL 80% methanol (−20 °C) followed by centrifugation at 4 °C for 10 min at 21,000*g*. The supernatant was dried in a SpeedVac system. MS buffer (2% methanol and 0.1% FA) was added to dissolve the dried fraction and standards which were then centrifuged for 10 min at 21,000*g* and the supernatants were transferred to liquid chromatography–mass spectrometry (LC–MS) vials. The LC–MS measurement was according to a previous study with some slight modifications.^32^ Briefly, 5 μL of the sample was injected into an Accucore Vanquish C18+ (100 × 2.1 mm, 1.5 μm particle size) UHPLC column, equipped with an Accucore Defender guards pk4 guard column (150 - C18 10 × 2.1 mm, 2.5 μm particle size (Thermo Fisher Scientific). The mobile phase system consisted of a mixture of solvent A (10 mM NH_4_OAc in mqH_2_O, pH 6.9) and solvent B (LC–MS grade methanol). A gradient elution method was used for the analysis: 0–1 min 5% B, 5–30 min linear gradient to 85% B, return to 0% B over 0.1 min, and kept for 10 min. The flow was kept constant at 0.25 mL/min, and the column temperature was kept at 30 °C throughout the analysis. MS analysis was performed in positive ion mode with the following parameters: Resolution, 120,000; spray voltage, 4 kV; capillary temperature, 350 °C; sheath gas, 35; auxiliary gas, 10. The full MS mass range was set at 100–1200 *m*/*z*. The collision energy for collision-induced dissociation (CID) was set at 35 eV. Xcalibur software (Thermo Fisher Scientific) and MS-DIAL v4.9 were used to analyze the data.

### 2.14 Statistical Analysis

All experiments were repeated in at least three biological independent replicates. All data were represented as the mean ± standard error of the mean (SEM). Microsoft Excel and GraphPad Prism v9 were used for all statistical analyses. The significant difference was calculated by the two-tailed unpaired student’s *t* test or one-way ANOVA with Tukey’s HSD post-test. The *p* values were indicated in the figure legends along with the statistical tests.

## 3 Results

### 3.1 Hst Inhibited PA-Induced Cell Death and NLRP3 Inflammasome Activation in HepG2 Cells

**Figure 1:**
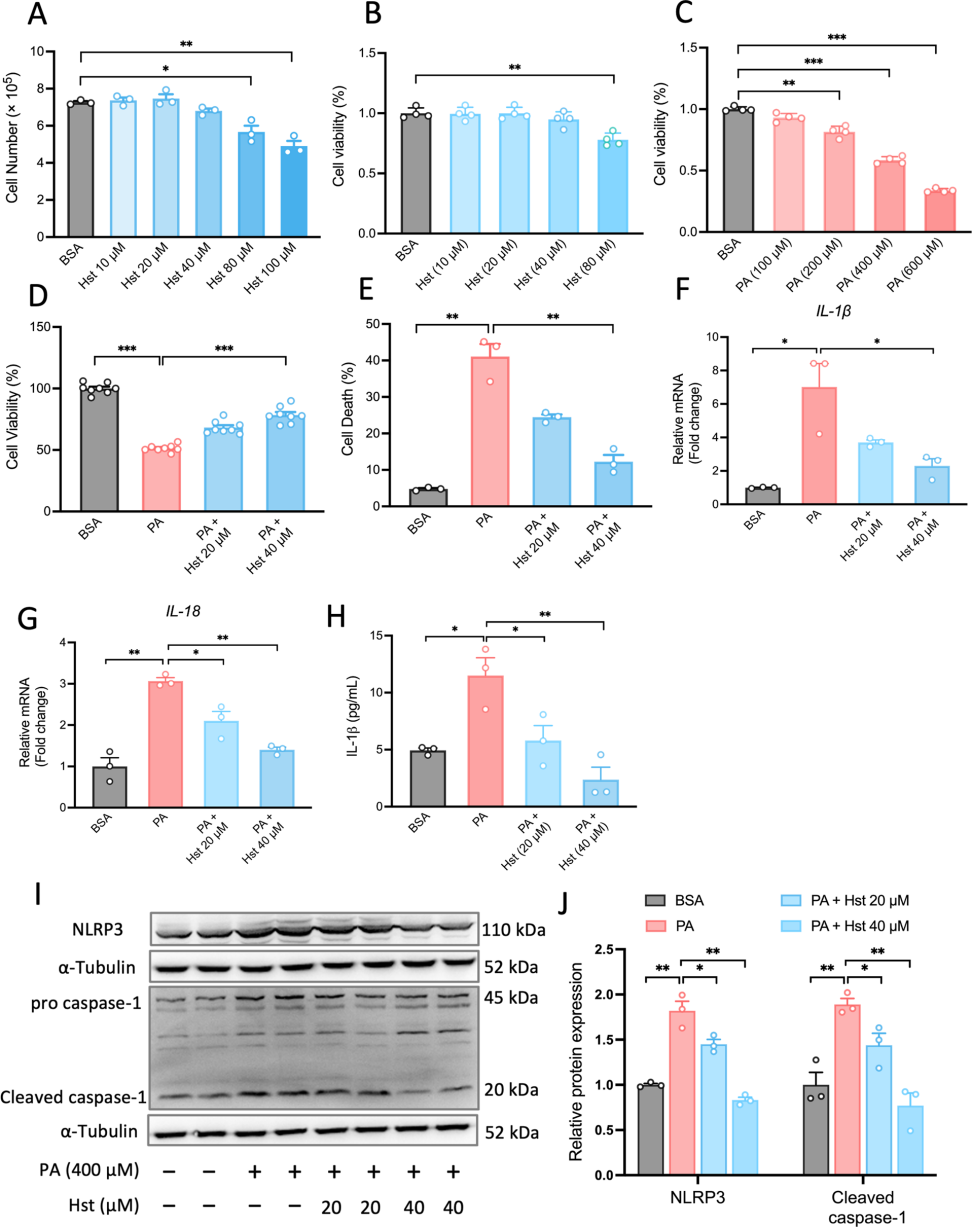
Hesperetin (Hst) attenuated palmitic acid (PA)-induced NLRP3 inflammasome activation in HepG2 cells. (A) Cells treated with Hst at indicated concentrations of 10, 20, 40, 80, and 100 μM for 24 h, the number of viable cells in HepG2 cells. (B,C) Cell viability of HepG2 cells incubated with Hst (10, 20, 40, and 80 μM), or PA (100, 200, 400, and 600 μM), respectively, for 24 h. HepG2 cells pretreated with or without Hst at concentrations of 20 and 40 μM for 4 h and then exposed to PA (400 μM) for 24 h. (D) Cell viability, (E) cell death, mRNA expression of (F) *IL-1β* and (G) *IL-18* measured by qPCR and (H) IL-1β secretion assessed by ELISA. (I) Representative immunoblotting and (J) quantification of NLRP3 and caspase-1 proteins. α-Tubulin was used as a loading control. Data are presented as mean ± SEM of *n* ≥ 3 independent experiments. *p < 0.05, **p < 0.01, ***p < 0.001 (unpaired two-tailed student’s *t* test).

In order to determine suitable concentrations of Hst and PA, we first explored its effects on the number of viable HepG2 cells using the trypan blue exclusion assay. HepG2 cells were incubated with Hst at a concentration range of Hst (10, 20, 40, 80, and 100 μM) for 24 h. The results indicated that at concentrations between 10 and 40 μM, Hst did not exhibit any observable toxic effects on HepG2 cells (Figure 1A). Similarly, Figure 1B shows that Hst (10, 20, and 40 μM) had no visible impact on cell viability measured by a CCK-8 kit. Furthermore, considering that PA at a concentration of 400 μM resulted in a 50% reduction in cell viability (Figure 1C), PA at a concentration of 400 μM, along with Hst at concentrations of 20 and 40 μM, were chosen for subsequent experiments. To test whether Hst prevents PA-induced cell death in HepG2 cells, we measured the percentage of cell death using a trypan blue exclusion assay and determined cell viability using a CCK-8 kit. We observed that PA treatment significantly increased cell death and decreased cell viability in HepG2 cells. Preincubation of HepG2 cells with Hst at concentrations of 20 and 40 μM increased cell viability as much as 17 and 27% relative to the PA group and decreased PA-induced cell death as much as 16 and 29%, respectively, suggesting its cytoprotective action in PA-treated HepG2 cells (Figure 1D,E). NLRP3 inflammasome activation in hepatocytes initiates excessive inflammatory responses and leads to subsequent hepatic death, contributing to the pathogenesis of NAFLD. To investigate whether the cytoprotective effect of Hst was related to the inhibition of NLRP3 inflammasome activation, the mRNA levels of *IL-1β* and *IL-18*, which are two downstream effectors of the NLRP3 inflammasome, were assessed. As presented in Figure 1F,G, Hst inhibited PA-induced *IL-1β* and *IL-18* mRNA expression in HepG2 cells. Consistent with these changes, the IL-1β secretion from HepG2 (Figure 1H), protein abundance of NLRP3 and cleaved caspase-1 (Figure 1I,J) were found to be repressed concentration-dependently in PA-incubated HepG2 cells upon Hst pretreatment. These data indicated that Hst attenuated PA-induced activation of the NLRP3 inflammasome, thereby preserving HepG2 cell viability.

### 3.2 Hst Improved PA-Induced Abnormal Metabolite and Energetic Profiles in HepG2 Cells

**Figure 2:**
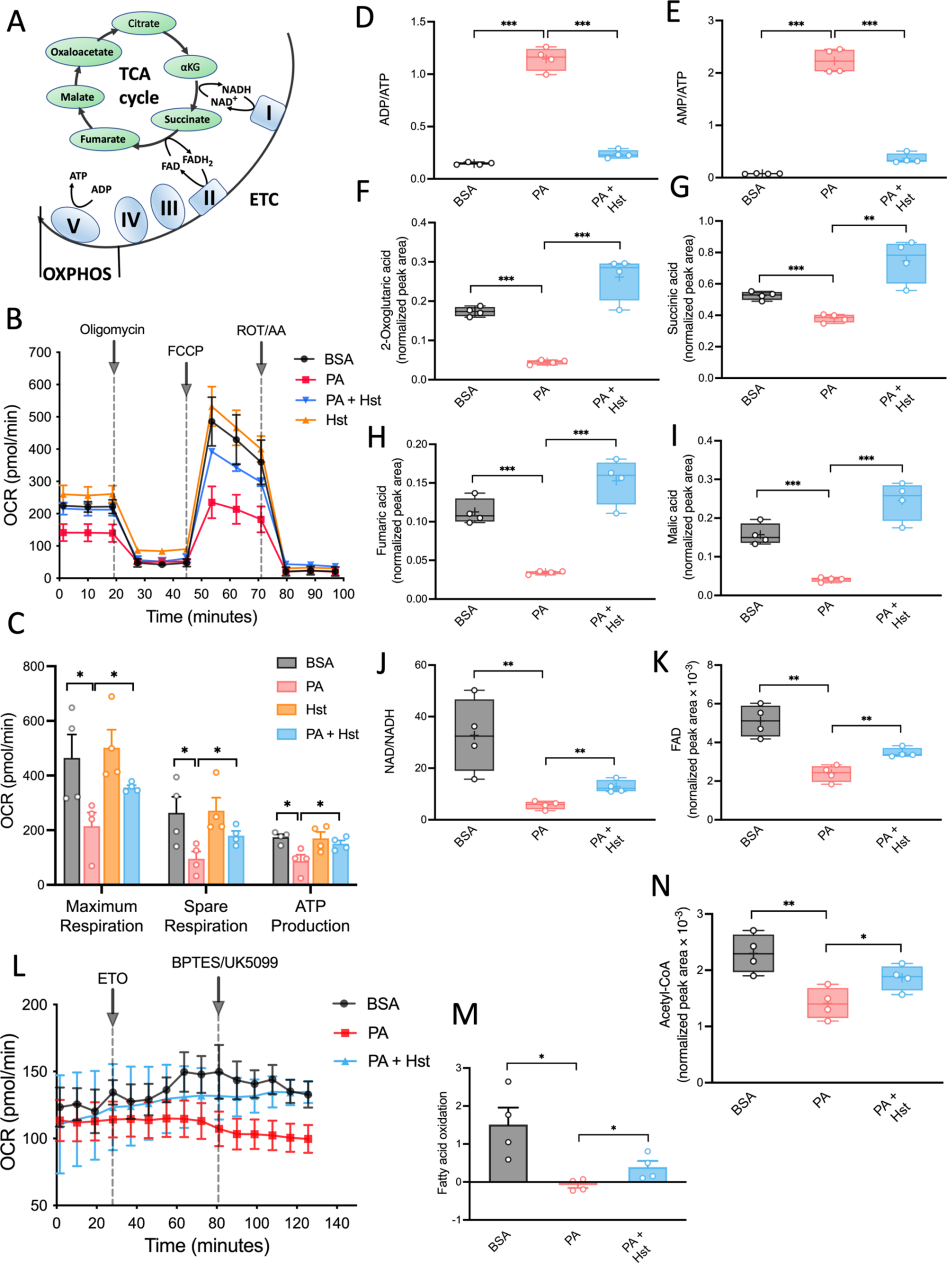
Hesperetin (Hst) elevated palmitic acid (PA)-induced abnormal mitochondrial metabolism in HepG2 cells. Cells were preincubated with Hst (40 μM) for 4 h and then exposed to PA (400 μM) for 24 h. (A) Schematics of the TCA cycle and OXPHOS. (B) Oxygen consumption (OCR) and (C) individual parameters for maximum respiration, spare respiration, and ATP production. (D,E) Ratio of ADP/ATP and AMP/ATP. (F–K) Intercellular levels of metabolites from the TCA cycle. MS peak areas were normalized to internal standards and corresponding pellet protein concentration. (L,M) Fatty acid dependency of OXPHOS was measured by a Seahorse Excellular Flux Analyzer. (N) Intercellular levels of acetyl-CoA. MS peak areas were normalized to internal standards and corresponding pellet protein concentration. Data are presented as mean ± SEM of *n* = 4 independent experiments. *p < 0.05, **p < 0.01, ***p < 0.001 (unpaired two-tailed student’s *t* test).

The central function of mitochondria is to produce cellular ATP. Mitochondrial ATP generation is accomplished by the electron transport chain utilizing electrons derived from the tricarboxylic acid cycle (TCA) cycle through OXPHOS (Figure 2A). To examine the impact of Hst on mitochondrial function in PA-treated HepG2 cells, we first measured OCRs. In comparison with control groups, PA-stimulated HepG2 cells exhibited a significant decrease in the maximal respiration, spare respiratory capacity, and OCR coupled to ATP production (Figure 2B,C). Hst effectively relieved the impaired mitochondrial bioenergetics induced by PA (Figure 2B,C). In line with the changes in mitochondrial OXPHOS efficiency, the incubation of HepG2 cells with Hst diminished the elevated ratio of ADP/ATP and AMP/ATP upon PA exposure (Figure 2D,E). Prompted by these results, we investigated whether Hst also had a beneficial effect on TCA cycle intermediates that were altered in response to PA exposure in HepG2 cells. Metabolomic analysis revealed that Hst obviously repressed the ability of PA to slow down the TCA cycle and blunt related TCA metabolites production including αKG, succinate, fumarate, and malate (Figure 2F–I). Furthermore, declined FAO has been recognized as a feature in NAFLD animal and cell models. We therefore examined the impact of Hst on PA-induced FAO rates by seahorse metabolic flux analyses. FAO, also termed mitochondrial β-oxidation, provides acetyl-coenzyme (acetyl-CoA) intermediates to the TCA cycle for the generation of NADH and FADH_2_. As shown in Figure 2L,M, PA exposure indeed resulted in a decreased rate of FAO and a reduction of NAD/NADH (Figure 2J) and FAD (Figure 2K), whereas Hst was effective in restoring FAO rate, FAD generation, and the ratio of NAD/NADH in PA-treated HepG2 cells. Notably, PA significantly decreased the level of acetyl-CoA, and Hst increased the acetyl-CoA level in HepG2 cells (Figure 2N).

### 3.3 Hst Suppressed Impairment of Mitochondria Dysfunction Caused by PA in HepG2 Cells

**Figure 3:**
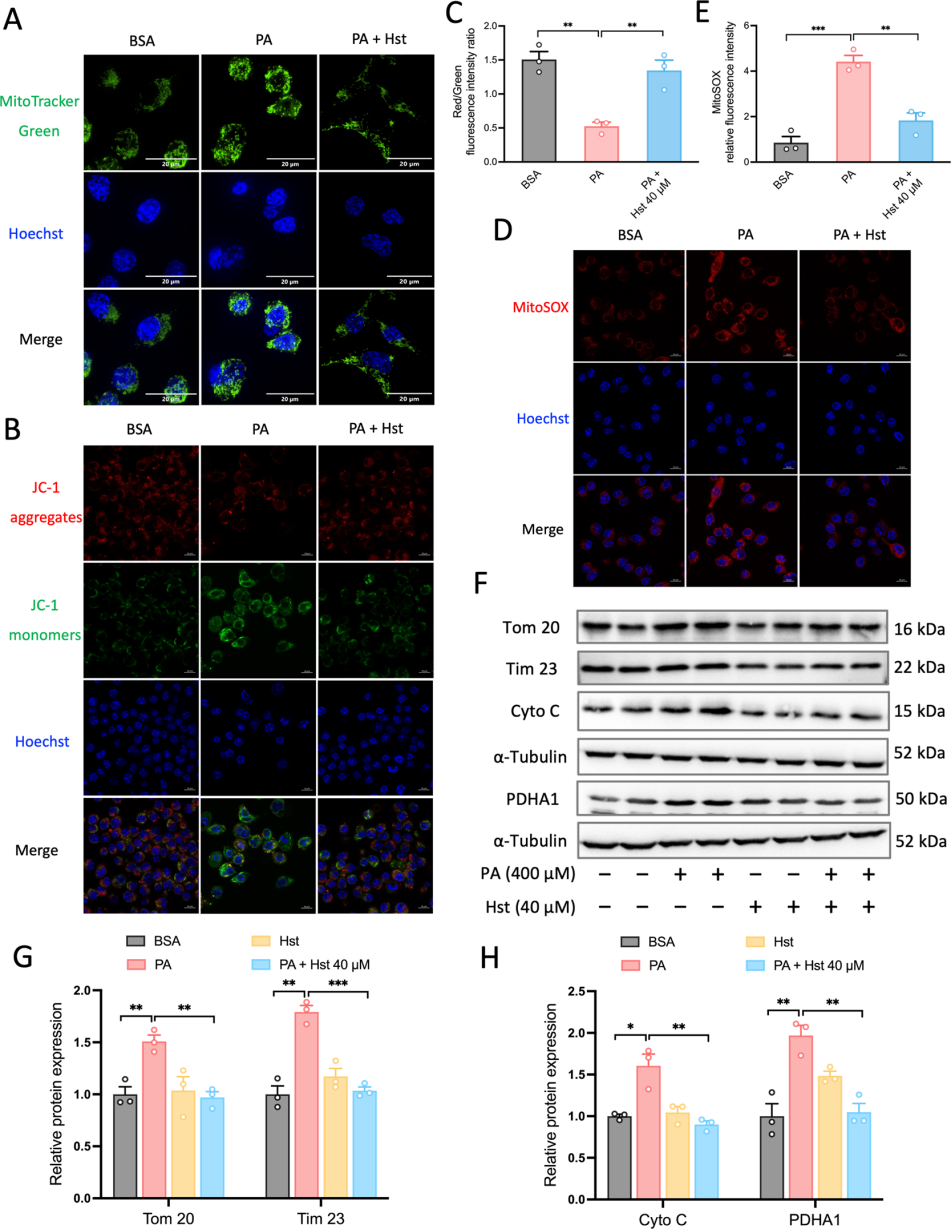
Hesperetin (Hst) reduced palmitic acid (PA)-elicited mitochondrial dysfunction and damaged mitochondria accumulation in HepG2 cells. Cells were pretreated with or without Hst at a concentration of 40 μM for 4 h prior to stimulation with PA (400 μM) for 24 h. (A) Cells were stained with MitoTracker Green and images were taken by spinning disk confocal microscopy. Nuclei were counterstained with Hoechst 33342 (blue). (B) Representative images and (C) quantification of mitochondrial membrane potential and mitochondrial ROS (D,E). (F) Representative immunoblotting and (G,H) quantification of the indicated mitochondrial proteins. α-Tubulin was used as a loading control. Nuclei were counterstained with Hoechst 33342 (blue). Data are presented as mean ± SEM of *n* = 3 independent experiments. *p < 0.05, **p < 0.01, ***p < 0.001 (unpaired two-tailed student’s *t* test). Scale bars are 20 μm in (A), 10 μm in (B,D).

Damaged mitochondria are consistently associated with morphological abnormalities. For this purpose, we used Spinning Disk confocal microscopy to observe the shape of the mitochondria. In PA-treated cells, mitochondria exhibited pronounced fragmentation compared to control cells, while Hst treatment reduced this fragmentation, as evidenced by live cell imaging analyses of mitochondria with MitoTracker Green (a fluorescent probe that localizes to mitochondria) (Figure 3A). We next studied its potential consequences on the mitochondrial membrane potential (ΔΨ_m_) by JC-1 dye staining. We found that PA-induced reduction of ΔΨ_m_ in HepG2 cells was overcome by Hst treatment, which was reflected by the elevation of the ratio of red/green intensities (Figure 3B,C). ΔΨ_m_ is an essential component for the ability of OXPHOS to drive the synthesis of ATP. Collapsed ΔΨ_m_ is a hallmark of mitochondrial dysfunction. Considering the strong correlation between ΔΨ_m_ and mitochondrial ROS generation, we next assessed mtROS levels by mitoSOX red staining, a cell-permeant fluorescent indicator of mtROS. Our results revealed that Hst suppressed mtROS production caused by PA, as evidenced from the decreased mitoSOX red intensities in Hst-treated HepG2 cells upon PA exposure (Figure 3D,E). Taken together, these data demonstrated that Hst restoring the mitochondrial functions disrupted by PA might contribute to inhibited inflammasome activation and reduced cell death.

### 3.4 Hst Reduced Accumulation of Damaged Mitochondria and Activated PINK1/Parkin-Mediated Mitophagy

**Figure 4:**
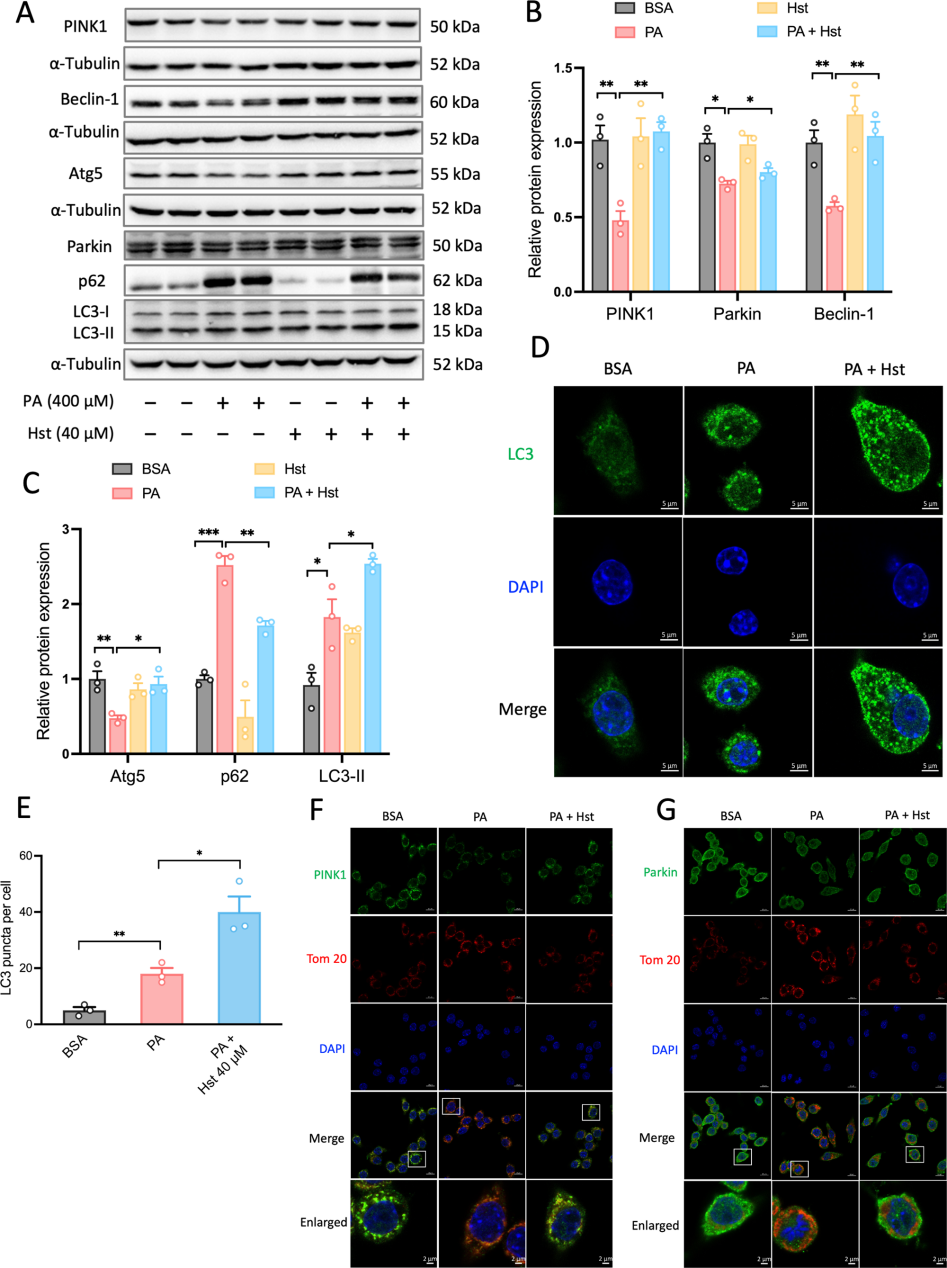
Hesperetin (Hst) restored palmitic acid (PA)-impaired mitophagy-mediated degradation in HepG2 cells. Cells were preincubated with Hst for 4 h at a concentration of 40 μM and then treated with or without PA (400 μM). (A) Representative immunoblotting and (B,C) quantification of the indicated proteins. α-Tubulin was used as a loading control. Cells were immunostained for LC3 (green). (D) Representative images and (E) quantification of the numbers of punctate LC3+ structures per cell. Representative immunostained images of (F) Tom 20 and PINK1, (G) Tom 20 and Parkin in HepG2 cells. Nuclei were counterstained with DAPI (blue). Data are presented as mean ± SEM of *n* = 3 independent experiments. *p < 0.05, **p < 0.01, ***p < 0.001 (unpaired two-tailed student’s *t* test). Scale bars are 5 μm in (D), and 10 μm in (F,G).

The improvement of mitochondrial function prompted us to determine whether the protection of Hst resulted from the removal of defective mitochondria. To test this, we analyzed the protein expression of the mitochondrial outer membrane (Tom 20), inner membrane (Tim 23), intermembrane (Cytochrome C), and matrix (PDHA1) by immunoblotting.^17^ As seen in Figure 3F–H, Hst treatment significantly reduced the increased abundance of these four mitochondrial proteins elevated by PA exposure in HepG2 cells. As damaged mitochondria are normally eliminated by mitophagy, we hypothesized that Hst diminished mitochondrial dysfunction occurring in PA-treated HepG2 cells by activating mitophagy-driven elimination of PA-impaired mitochondria. To verify this, we detected autophagy by monitoring the LC3 conversion by immunoblotting and immunofluorescence. The conversion of the nonlipidated form of LC3-I to the lipidated form of LC3-II is an indicator of autophagic flux. Compared to PA-exposed cells, we noticed a further elevated ratio of LC3-II/LC3-I (Figure 4A,C) and higher numbers of LC3-positive puncta (Figure 4D,E) in HepG2 cells incubated with Hst. The induction of autophagy by Hst was substantiated by the repression of increased protein levels of p62 elicited by PA in HepG2 cells (Figure 4A,C). Moreover, improved protein expressions of Beclin-1 and Atg5 were observed in HepG2 cells incubated with Hst upon PA stimulation (Figure 4A–C), which are involved in the formation of autophagosomes and assistance of LC3 lipidation. This was paralleled by the elevation of PINK1 and Parkin, the key drivers of mitophagy, in PA-treated HepG2 cells (Figure 4A,B). To visualize the specific effect of Hst on PINK1 and Parkin cellular localization and mitochondria simultaneously, we conducted co-immunofluorescence staining of PINK1 and Tom 20 as well as Parkin and Tom 20, respectively. As shown in Figure 4F,G, the co-localization of PINK1 and Tom 20, Parkin and Tom 20 in PA-treated HepG2 cells was decreased compared to control cells. Hst incubation resulted in an increased overlap of PINK1 and Tom 20, or Parkin and Tom 20 in PA-stimulated cells.

### 3.5 Hst Restrained PA-Induced NLRP3 Inflammasome Activation and Relieved Mitochondrial Dysfunction through Promoting Mitophagy

**Figure 5:**
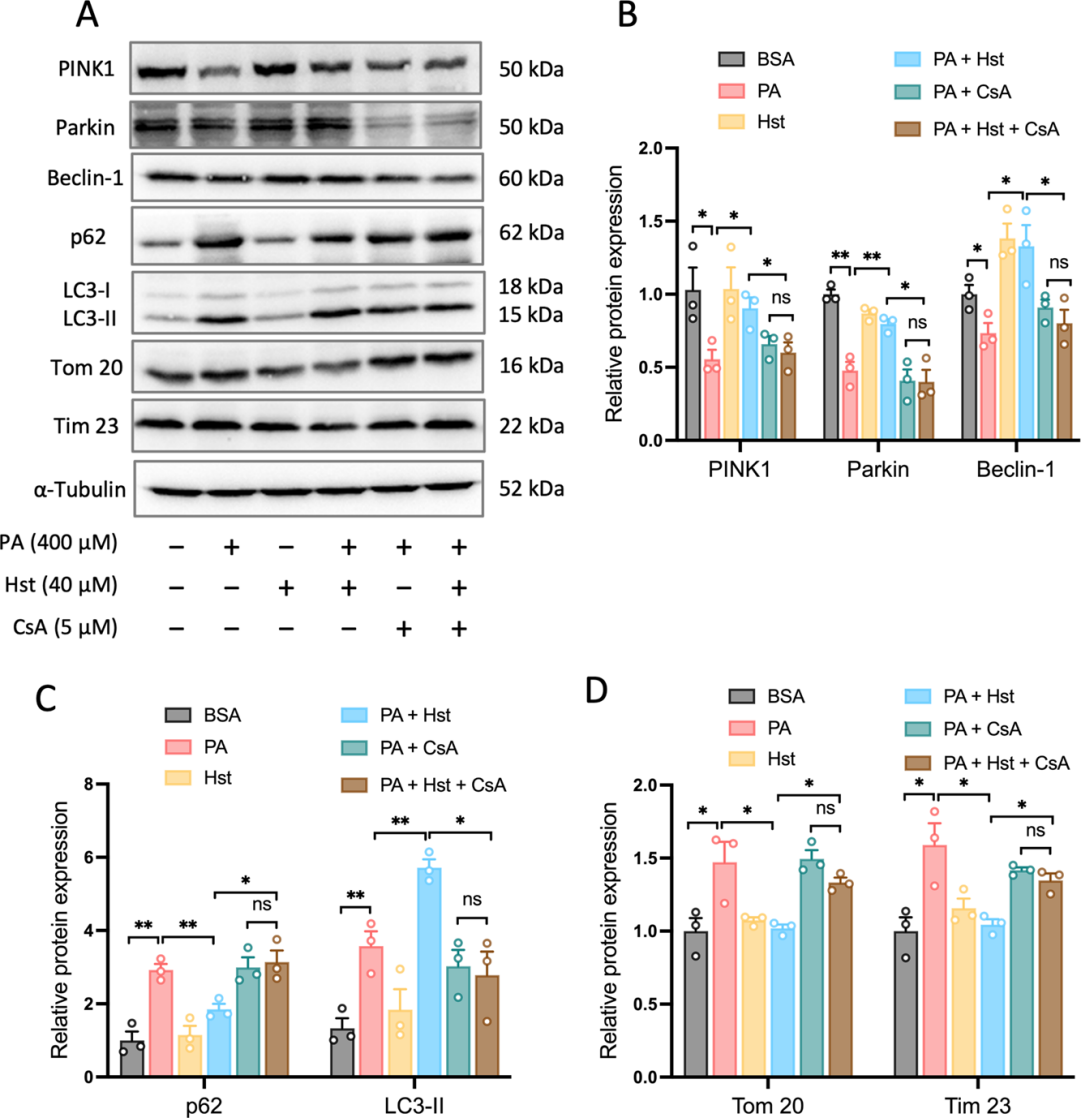
Treatment with cyclosporin (CsA) decreased Hesperetin (Hst)-induced PINK1-driven mitophagy in palmitic acid (PA)-stimulated HepG2 cells. Cells were pretreated with or without CsA (5 μM) for 4 h in the absence or presence of 40 μM Hst before the addition of PA (400 μM). (A) Representative immunoblotting and (B–D) quantification of the indicated proteins. Data are presented as mean ± SEM of *n* = 3 independent experiments. *p < 0.05, **p < 0.01, ***p < 0.001 (unpaired two-tailed student’s *t* test).

**Figure 6:**
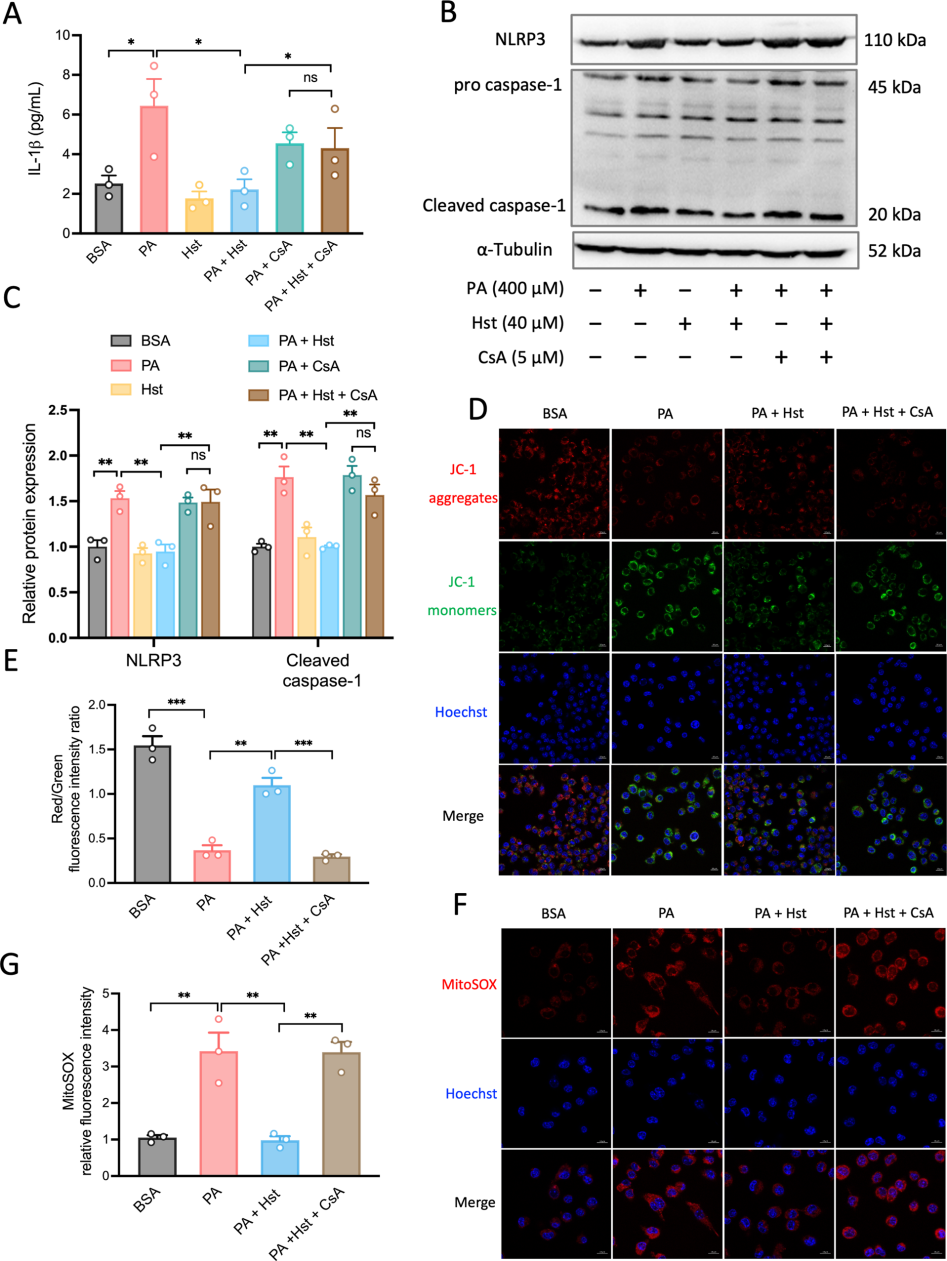
Inhibition of mitophagy by cyclosporin (CsA) blunted the protective effect of Hesperetin (Hst) against palmitic (PA)-triggered NLRP3 inflammasome activation and mitochondrial dysfunction in HepG2 cells. Cells were pretreated with or without CsA (5 μM) for 4 h in the absence or presence of 40 μM of Hst before addition of PA (400 μM). (A) IL-1β production determined by ELISA assay. (B) Representative immunoblots and (C) quantification of NLRP3 and caspase-1. α-Tubulin was used as a loading control. Cells were loaded with JC-1 (mitochondrial membrane potential fluorescent probe, 2 μM) or with mitoSOX (red, mitochondrial ROS indicator, 1 μM), and analyzed by confocal microscope. Representative images and quantification of (D,E) mitochondrial membrane potential and (F,G) mitochondrial ROS. Nuclei were counterstained with Hoechst 33342 (blue). Data are presented as mean ± SEM of *n* = 3 independent experiments. *p < 0.05, **p < 0.01, ***p < 0.001 (unpaired two-tailed student’s *t* test). Scale bars are 10 μm.

To corroborate the role of mitophagy in Hst-mediated inhibition of PA-elicited activation of NLRP3 inflammasome, we exposed HepG2 cells to cyclosporin A (CsA), a well-defined inhibitor of mitochondrial cyclophilin D, which is extensively used to inhibit mitophagy.^33−36^ The results indicated that CsA treatment prevented the augmentation of mitophagy by Hst, as evidenced by the reduction of PINK1, Parkin, and Beclin-1 protein expressions (Figure 5A,B), p62 accumulation (Figure 5A,C), and LC3-I-to-LC3-II conversion (Figure 5A,C) as well as downregulation of Tom 20 and Tim 23 (Figure 5A,D) in PA-treated HepG2 cells. In addition, CsA abolished the ability of Hst to decrease IL-1β production, NLRP3, and cleaved caspase-1 in PA-treated HepG2 cells (Figure 6A–C). Then, we assessed the impacts of CsA on the release of mtROS and ΔΨ_m_ in PA-stimulated HepG2 cells upon Hst incubation. As seen in Figure 6D–G, treatment with CsA exacerbated mitochondrial alterations, including decreased ΔΨ_m_ and increased mtROS to a greater extent in PA-stimulated HepG2 cells incubated with Hst compared to Hst-untreated cells.

### 3.6 Silencing *Pink1* Impaired the Beneficial Effects of Hst

**Figure 7:**
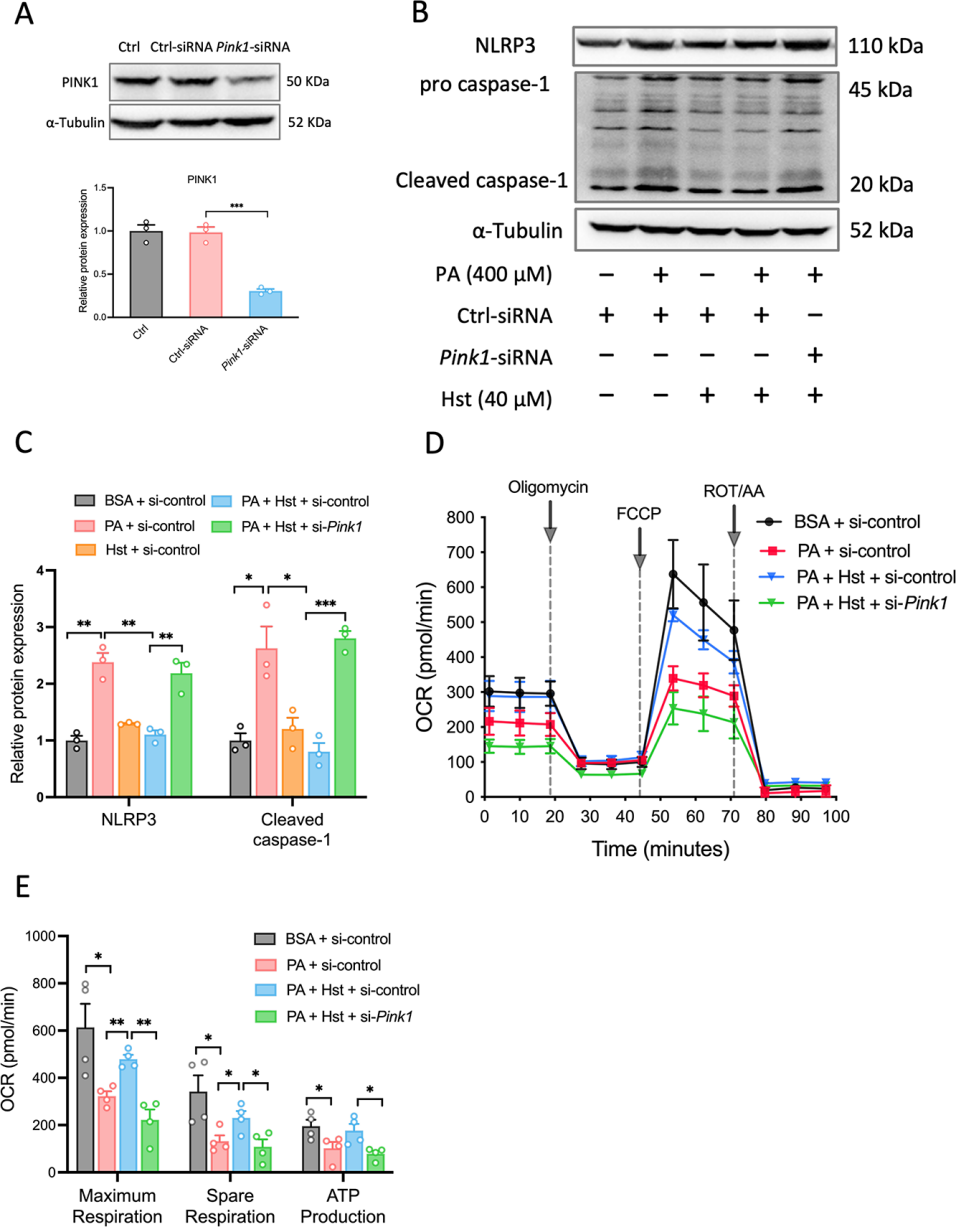
Suppression of NLRP3 inflammasome activation and mitochondrial dysfunction by Hesperetin (Hst) is mediated by PINK1 in HepG2 cells. Cells were transduced with control-siRNA or *Pink1*-siRNA, and then pretreated with or without 40 μM Hst for 4 h in the presence or absence of 400 μM palmitic acid (PA). (A) Representative immunoblotting and quantification of PINK1 protein expression. (B) Representative immunoblotting and (C) quantification of NLRP3 inflammasome. (D) Oxygen consumption (OCR) and (E) individual parameters for maximum respiration, spare respiration, and ATP production. Data are presented as mean ± SEM of *n* ≥ 3 independent experiments. *p < 0.05, **p < 0.01, ***p < 0.001 (unpaired two-tailed student’s *t* test).

**Figure 8:**
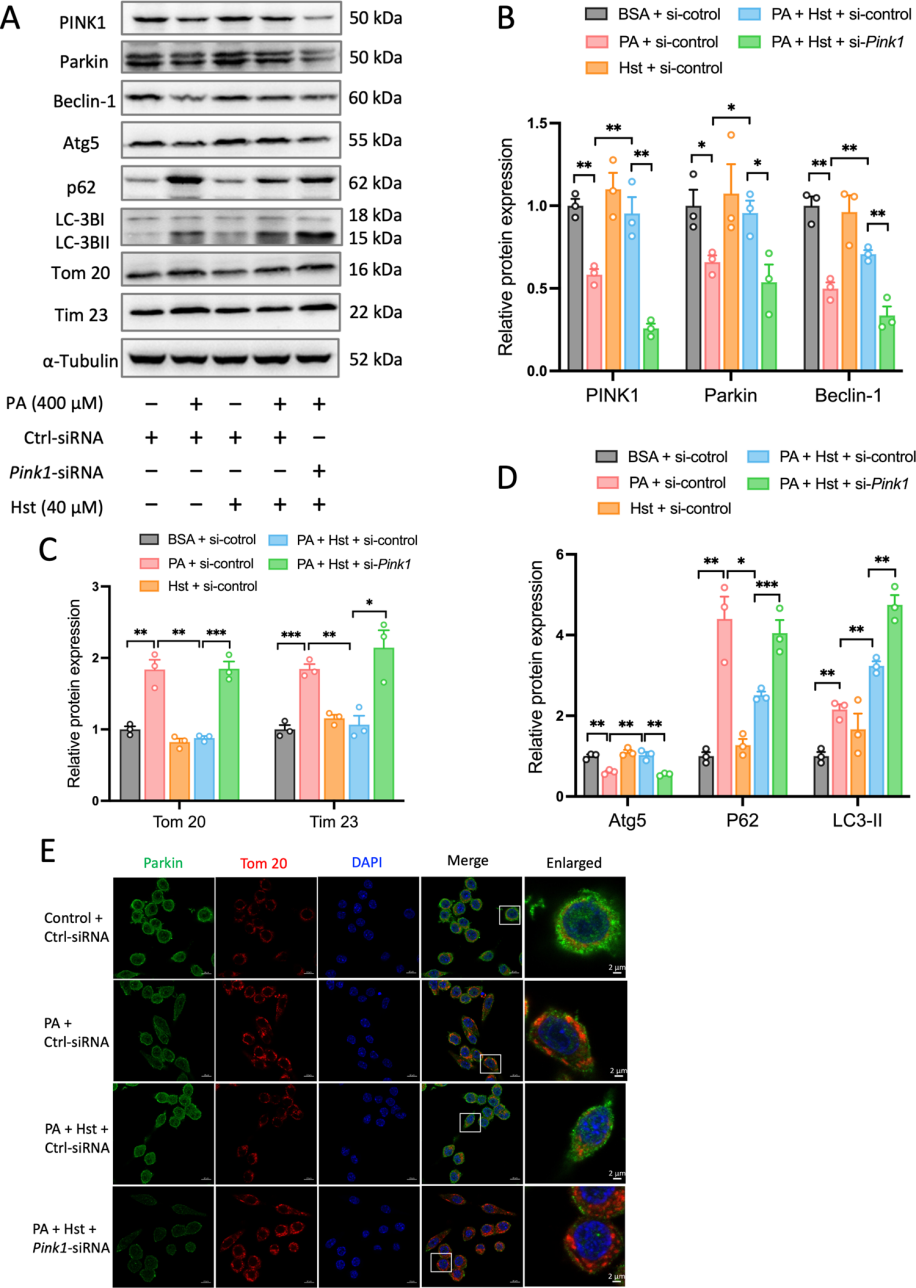
PINK1-directed mitophagy involved preventive effects of Hesperetin (Hst) in palmitic acid (PA)-treated HepG2 cells. Cells were transduced with control-siRNA or *Pink1*-siRNA, and then preincubated with or without 40 μM Hst for 4 h prior to exposure to 400 μM of PA. (A) Representative immunoblotting and (B–D) quantification of indicated proteins. Representative immunostained images of (E) Parkin and Tom 20 in HepG2 cells. Nuclei were counterstained with DAPI (blue). Data are presented as mean ± SEM of *n* = 3 independent experiments. *p < 0.05, **p < 0.01, ***p < 0.001 (unpaired two-tailed student’s *t* test). Scale bars, 10 μm.

As PINK1 has been proven to be the key driver of mitophagy, we further determined the extent to which it contributes to the preventive effects of Hst on NLRP3 inflammasome activation and mitochondrial dysfunction in PA-treated HepG2 cells. To achieve this, we transfected HepG2 cells with *Pink1* siRNA to inhibit the PINK1 protein expression. As shown in Figure 7A, *Pink1*-siRNA reduced the PINK1 protein expression by about 70%, indicating a successful knockdown. Suppression of *Pink1* disrupted the protection of Hst against PA-triggered NLRP3 inflammasome activation in HepG2 cells, as indicated by the significant increased protein expressions of NLRP3 and cleaved caspase-1 (Figure 7B,C). Importantly, *Pink1* silencing blunted the impact of Hst on impaired mitochondrial bioenergetics induced by PA exposure in HepG2 cells, as evidenced by the significant reduction of the total respiratory capacity, ATP turnover, and spare respiratory capacity (Figure 7D,E). Furthermore, *Pink1* silencing impinged on the ability of Hst to increase Parkin, Beclin-1, and Atg5 protein expression levels in PA-treated HepG2 cells (Figure 8A,B). Similarly, inhibition of *Pink1* overcame the impact of Hst on the conversion of LC3-II to LC3-I (Figure 8A,D) and reversed the ability of Hst in decreasing the accumulation of p62, Tom 20, and Tim 23 proteins in PA-stimulated HepG2 cells (Figure 8A,C,D). Accordingly, the co-localization of Parkin and Tom 20 in PA-incubated HepG2 cells was blunted by *Pink1* silencing (8E). These results demonstated that PINK is necessary for the protective effects of Hst in PA-stressed hepatocytes.

## 4 Discussion

The hepatocytes are enriched with mitochondria to maintain their specialized functions and cellular integrity. Defects that disrupt mitochondrial homeostasis lead to massive hepatocyte death and loss of liver function, largely affecting the development of NAFLD.^37,38^ Mitochondrial dysfunction triggered by PA has been described as a causative factor of liver failure in NAFLD. Thus, supporting mitochondrial function is of the utmost importance in the control of liver injury for NAFLD treatment. In this study, HepG2 cells were stimulated with PA working as a high-fat model.^6,39^ Our results found that Hst exerts protective effects in PA-treated HepG2 cells, notably by modulating the PINK1/Parkin-driven mitophagy and preserving energetic metabolism, mitochondrial metabolic plasticity, as well as overall mitochondrial function.

In conditions of PA overload, hepatocytes exhibit reduced ΔΨ_m_, suppressed mitochondrial respiration and ATP production.^7^ Of note, these mitochondrial defects are reversed by incubating HepG2 cells with Hst, demonstrating the role of Hst in preventing PA-elicited mitochondrial dysfunction. Furthermore, Hst reduced the heightened mtROS levels in PA-treated HepG2 cells. Excessive ROS released from electron transport chain (ETC) during OXPHOS have been considered to induce damage to cellular membranes, proteins, and DNA, especially to those regulatory enzymes within the TCA cycle that are highly vulnerable to oxidative damage, which results in TCA cycle shutdown.^40,41^ Here, we provide evidence that Hst increased most of the TCA cycle intermediates including αKG, succinate, fumarate, and malate. It is tempting to speculate that the action of Hst on PA-induced TCA cycle shutdown is mediated by buffering mtROS overproduction. Beyond providing biosynthetic intermediates for macromolecule production, the TCA cycle contributes to the generation of ATP. Two reducing equivalents NADH and FADH_2_ that derive from the TCA cycle donate electrons to drive the ETC for ATP synthesis.^41,42^ The restoration of the TCA cycle function by Hst partly explains its facilitating effects on the OXPHOS and ATP content. Moreover, there is strong evidence that increasing NAFLD severity corresponded with a loss in hepatic FAO.^4,43,44^ Intriguingly, by using fuel dependency assays and LC-MS analysis, our findings revealed that treatment with Hst increased FAO rates and intercellular acetyl-CoA level in PA-treated HepG2 cells, responsible for supplying the TCA cycle with additional NADH and FADH_2_ being fed into the ETC.^45^ These findings highlight the important role that Hst plays in regulating mitochondrial function and shed light on the potential benefit of Hst in regulating mitochondrial metabolism.

In addition to energy and metabolism, mitochondria play a crucial role in regulating some key cellular processes, such as apoptosis and inflammation. A growing body of literature suggests that excessive ROS generated by defective mitochondria can result in the activation of NLRP3 inflammasome.^20,46^ Recent studies have indicated the associations of PA-induced hepatocyte death with dysregulated inflammasome. NLRP3 inflammasome comprising multiple proteins could be activated by a wide variety of danger signals and provoke innate immune responses.^8,9^ Assembly of the NLRP3 inflammasome activates caspase-1 via initiating cleavage of procaspase 1 into caspase-1, which subsequently converts the cytokine precursors pro-IL-1β into mature and biologically active IL-1β.^47^ The release of mature IL-1β mediates many immune reactions.^48^ Consistently, our results show that Hst could alleviate PA-triggered cell death by limiting mitochondrial dysfunction and regulating NLRP3 inflammasome activation induced by PA in HepG2 cells.

We observed accumulation of damaged mitochondria in PA-stimulated HepG2 cells due to deficient mitophagy. The accumulation of dysfunctional mitochondria leads to increasing mtROS concentrations, disrupts redox homeostasis, and causes oxidative stress.^49^ Oxidative stress is thought to be the direct cause in the development of NAFLD.^4,5^ To cope with mitochondrial damage, cells make use of a catabolic self-eating process termed autophagy to constantly degrade defective mitochondria to sustain a proper population of mitochondria within the cells.^17,50^ Sufficient mitophagy plays a major role in removing the damaged mitochondria and maintaining cellular homeostasis.^51^ Our results demonstrated that Hst-mediated improvements in mitochondrial function and cell survival can likely be attributed to mitophagy stimulation. Interestingly, recent work revealed that mitophagy enhancement inhibits PA-induced mitochondrial stress and alleviates the progression of NAFLD.^52,53^ Given these studies, in addition to our own, mitophagy induction should be considered as an important mechanism for ameliorating NAFLD.

There is strong evidence that exhausted mitochondria could be identified and selectively eliminated by the PINK1/Parkin-dependent autophagy.^13,14^ In the present work, we show that Hst upregulated PINK1 protein expression and promoted co-localization of PINK1 and mitochondria. PINK1 acting as a molecular sensor detects signals of abnormal mitochondria for the recruitment and activation of Parkin. Through its E3 ubiquitin ligase activity, Parkin binds to the outer mitochondrial membrane, initiating multiple signaling events culminating in the engulfment of damaged mitochondria within lysosomes.^10,54^ It has been shown that loss of PINK1 impairs mitophagy and that disabled mitophagy is one of the potential contributors to the onset of NAFLD.^55,56^ In support of this, some studies reported inhibited PINK1/Parkin-mediated mitophagy in the livers of mice with NAFLD and PA-treated hepatocytes.^6,20^ Conversely, restoring PINK1/Parkin-driven mitophagy rescued the mitochondrial dysfunction and preserved cellular integrity and homeostasis in hepatocytes. Here, our results connect the hepatoprotective effects of Hst to PINK1/Parkin-mediated mitophagy upregulation. Moreover, these findings also support the role of PINK1 in maintaining the mitochondrial quality control system.

In conclusion, our results demonstrated the potential benefits of Hst in PA-treated HepG2 cells. We elucidate the molecular mechanism through which the action of Hst is linked to the inhibition of NLRP3 inflammasome, namely, by activation of the perturbed PINK1/Parkin-mediated mitophagy. By priming the mitophagy-mediated clearance and quality control system, Hst eliminated the accumulation of defective mitochondria in PA-treated HepG2 cells and subsequently led to increased mitochondrial function, including increased ΔΨ_m_ and ATP content, restored OXPHOS efficiencies, FAO rates, and increased TCA cycle intermediates and reducing equivalents, as well as normalized mtROS production. The elevation of properly functioning mitochondria attenuates the activation of the NLRP3 inflammasome, thereby lessening generated hepatic stress and cell damage. These findings not only uncover a novel pathway of actions of Hst in the prevention of NAFLD but also offer a promising therapeutic strategy for intervention against other mitochondrial disorders.
